# Axial Compression Behavior of Bamboo Scrimber-Filled Steel Tubular (BSFST) Column Under Different Loading Modes

**DOI:** 10.3390/ma18153607

**Published:** 2025-07-31

**Authors:** Ze Xing, Yang Wei, Kang Zhao, Jinwei Lu, Baoxing Wei, Yu Lin

**Affiliations:** 1 College of Civil Engineering, Nanjing Forestry University, Nanjing 210037, China; xingze@njfu.edu.cn (Z.X.); ljw99@njfu.edu.cn (J.L.); 13776629792@163.com (B.W.); linyuzero05@njfu.edu.cn (Y.L.); 2 Jiangsu Carbon Sequestration Materials and Structural Technology of Bamboo & Wood Research Center, Nanjing Forestry University, Nanjing 210037, China

**Keywords:** bamboo scrimber, bamboo scrimber-filled steel tubular column, axial compression, loading mode, calculation model

## Abstract

Bamboo scrimber is an environmentally friendly biomass building material with excellent mechanical properties. However, it is susceptible to delamination failure of the transverse fibers under compression, which limits its structural performance. To address this problem, this study utilizes steel tubes to encase bamboo scrimber, forming a novel bamboo scrimber-filled steel tubular column. This configuration enables the steel tube to provide effective lateral restraint to the bamboo material. Axial compression tests were conducted on 18 specimens, including bamboo scrimber columns and bamboo scrimber-filled steel tubular columns, to investigate the effects of steel ratio and loading mode (full-section and core loading) on the axial compression performance. The test results indicate that the external steel tubes significantly enhance the structural load-bearing capacity and deformation capacity. Primary failure modes of the composite columns include shear failure and buckling. The ultimate stress and strain of the structure are positively correlated with the steel ratio; as the steel ratio increases, the ultimate stress of the specimens can increase by up to 19.2%, while the ultimate strain can increase by up to 37.7%. The core-loading specimens exhibited superior load-bearing capacity and deformation ability compared to the full-section-loading specimens. Considering the differences in the curves for full-section and core loading, the steel tube confinement coefficient was introduced, and the predictive models for the ultimate stress and ultimate strain of the bamboo scrimber-filled steel tubular column were developed with accurate prediction.

## 1. Introduction

Bamboo has broad engineering applications due to its many advantages such as abundant availability, energy efficiency, environmental friendliness, and economic value, and it can serve as a complementary material to steel and concrete [1,2]. However, natural bamboo suffers from inherent defects such as dimensional variability, susceptibility to cracking, and poor durability [3,4]. To meet the demands of more complex engineering applications, various bamboo-based composites have been developed through modern processing technologies [5]. Among them, bamboo scrimber, a high-value-added product independently developed in China, is produced through milling, drying, gluing, and hot-pressing processes with a form of mainly boards [6]. This material not only offers superior toughness but also exhibits greater compressive and tensile strength than ordinary wood, enabling it to meet structural performance requirements [7,8].

In recent years, researchers have conducted extensive studies on the mechanical properties of bamboo scrimber [9,10]. Wei et al. [11] investigated the axial tensile and compressive properties of bamboo scrimber in detail and proposed an innovative axial tensile stress–strain relationship model. Building on this, Wei et al. [12] were the first to reveal the mechanical behavior of short bamboo scrimber columns under cyclic compressive loading. Three primary corresponding failure modes are defined, namely flexural instability, shear failure, and interfacial cracking. The specimens exhibited superior residual deformation capacity compared to conventional concrete in the elastic–plastic phase. A multi-stage coupled modeling approach was adopted to develop a numerical model capable of capturing the entire cyclic loading process. Hong et al. [13] conducted experiments on the eccentric compressive behavior of chamfered bamboo scrimber columns and found that specimens with varying eccentricities exhibited brittle tensile failure. They proposed an empirical equation for the ultimate load capacity, accounting for the effects of slenderness ratio and eccentricity. Mashrah [14] conducted a comprehensive comparative analysis of two important engineering bamboo forms: laminated bamboo lumber (LBL) and bamboo scrimber (BS). They examined their economic benefits, manufacturing processes, mechanical properties, failure modes, thermal behavior, and applications. Bamboo scrimber generally outperforms LBL in tensile, compressive, and flexural strengths. Both materials show degradation at high temperatures, but bamboo scrimber performs better under fire conditions. However, compressive loading of bamboo scrimber often results in transverse fiber delamination, which limits the full development of its mechanical performance [1].

To address the issue of limited compressive performance and transverse fiber delamination in bamboo scrimber, steel–bamboo composite structures have attracted increasing attention in recent years. These composite systems can not only significantly enhance the bearing capacity and utilization efficiency of bamboo but also mitigate premature local buckling of steel components and improve the overall structural stability. Zhang et al. [15] combined cold-formed thin-walled steel with bamboo to form a steel–bamboo composite structure, investigating the failure modes and interfacial mechanisms and analyzing the shear stress distribution at the bonding interface. Building on this, Zhang et al. [16] further investigated the mechanical behavior of the novel steel–bamboo composite columns under long-term loading. Zhao et al. [17] developed a novel hollow composite column composed of thin-walled steel pipes, bamboo plywood, and restraining steel reinforcements. The observed failure modes of bamboo plywood included crushing failure, partial debonding at the interface, and flexural instability. Their findings revealed that the ultimate bearing capacity of the column was influenced by factors such as net cross-sectional area, slenderness ratio, and the presence of confinement reinforcement. Building on this, Zhou et al. [18] further developed a bamboo plywood and thin-walled steel tube dual-confined concrete column (BSDCC) by pouring concrete into the steel tube and studied its compressive capacity. The results showed that the contribution of concrete to the bearing capacity was less than 10%, but it effectively alleviated the cracking failure of bamboo plywood and the buckling of the steel tube. Jin et al. [19] investigated the axial compressive behavior of twining-bamboo-confined thin-walled steel tubular (TBCST) columns and, through finite element simulation, it was concluded that the optimal thickness of the wrapped bamboo is 10 mm and the optimal winding angle is 75°. Gan et al. [20] proposed a new type of hollow composite column, which consists of an outer steel tube, an inner hollow bamboo tube, and a concrete cast between the inner and outer layers. Compared with concrete-filled double skin steel tubular (CFDST) columns, this structure uses environmentally friendly bamboo materials to replace the inner steel tube and exhibits excellent ductility and compressive capacity.

In another innovative approach of confining bamboo with steel tubes, Zhang et al. [21] proposed a new type of steel–bamboo–concrete composite column, where concrete was poured in the annular space between an outer steel tube and an inner raw bamboo core. The combined axial compression behavior of such composite short columns demonstrated notable synergistic effects among the three materials. Drawing inspiration from partially encased composite columns widely used in structural engineering, some scholars have developed partially wrapped bamboo scrimber structures. Dong et al. [22] proposed novel partially encased bamboo scrimber (PEBS) columns in which bamboo scrimber was embedded within the flanges of H-section steel beams. They carried out axial compression tests on composite columns and four-point bending tests on composite beams. The results showed significant improvements in both load-bearing and deformation capacity. The embedded bamboo also helped to reduce local buckling and improve lateral stability, while markedly enhancing the overall ductility of the structure. However, the synergy between steel and bamboo relies on a reliable and stable bonding interface. Additionally, local buckling of the steel is a major factor that undermines this synergy.

Therefore, the ideal steel–bamboo composite should prevent interfacial bond failure and premature local buckling. To address these challenges, a novel bamboo-scrimber-filled steel tubular (BSFST) column was proposed, in which the steel tube fully wraps the bamboo, providing it with uniform circumferential confinement. The steel tube effectively constrained the transverse expansion and cracking of the bamboo, forming a unified and stable compression boundary. This design mitigated premature interfacial failure, alleviated buckling and splitting issues in the bamboo, and significantly enhanced the overall structural performance. Axial compression tests on 18 BSFST columns and bamboo scrimber columns were conducted for comparison, and the impacts of steel ratio and on the failure modes, stress–strain relationship curves, and mechanical properties were analyzed. Furthermore, a method for calculating the ultimate stress and strain of BSFST columns was developed and verified.

## 2. Material and Methods

### 2.1. Specimen Design

**Figure 1:**
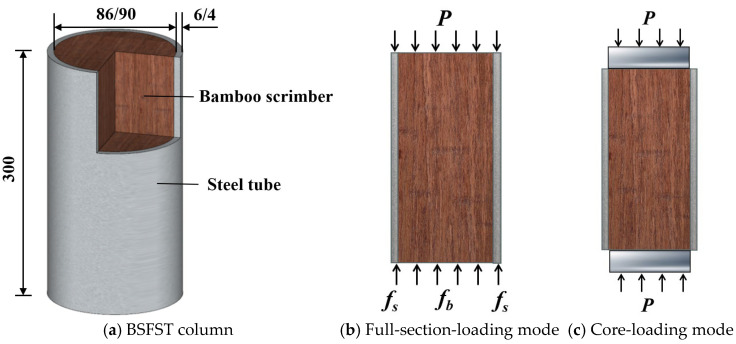
Structural concept diagram of bamboo scrimber-filled steel tubular column (unit: mm).

A total of 12 bamboo-scrimber-filled steel tubular columns and 6 control bamboo scrimber specimens were prepared for this experiment. As shown in Figure 1, Each composite column had a diameter of 100 mm and a height of 300 mm. The main variables were the loading mode (full-section or core loading) and the steel tube thickness (4 mm or 6 mm), resulting in four test groups. In the full-section-loading mode, both the steel tube and the bamboo were subjected to vertical loads. In the core-loading mode, only the bamboo core was loaded. To evaluate the confinement effect of the steel tube on the bamboo, the dimensions of the bamboo scrimber column and the bamboo core in the composite column were kept identical. Detailed parameters are listed in Table 1.

To clearly distinguish the specimens, a naming convention based on their parameters was adopted. Capital letters B, D, T denote the diameter of the bamboo specimen, the diameter of the composite column, and the steel tube thickness, respectively. F and C stand for full-section loading and core loading, respectively. Three specimens were prepared for each parameter group and distinguished using suffixes “-1”, “-2”, and “-3”. For example, “FD100T4-1” represents the first full-section-loaded composite column with a diameter of 100 mm and a steel tube thickness of 4 mm.

### 2.2. Material Properties

Steel tubes made of common carbon steel Q235 were used in the test. Three standard tensile specimens of each size were designed. As shown in Table 2, the corresponding tensile strength, average yield strength, and elastic modulus were measured according to Chinese Code (GB/T 228.1−2010) [24]. The mechanical properties of the bamboo scrimber employed in this study are shown in Table 3, which was made from Moso bamboo grown in Xuan Cheng, China. To comprehensively assess its mechanical properties, a total of fifteen specimens for tensile testing and fifteen for compressive testing were prepared with standardized dimensions and subjected to evaluation in accordance with ASTM D143-09, 2014 [25].

This test utilizes epoxy resin adhesive produced by Nanjing Mankate Company, Nanjing, China. The epoxy resin adhesive consists of two components: A and B, which are mixed in a ratio of 2:1 prior to use. According to the factory test report, the adhesive has a density of 1.15 g/cm^3^, a gel time of approximately 3 to 8 h, a compressive strength of 79.6 MPa, and a tensile strength of 49.8 MPa, with a tensile modulus of elasticity of 3200 MPa.

### 2.3. Test Program

**Figure 2:**
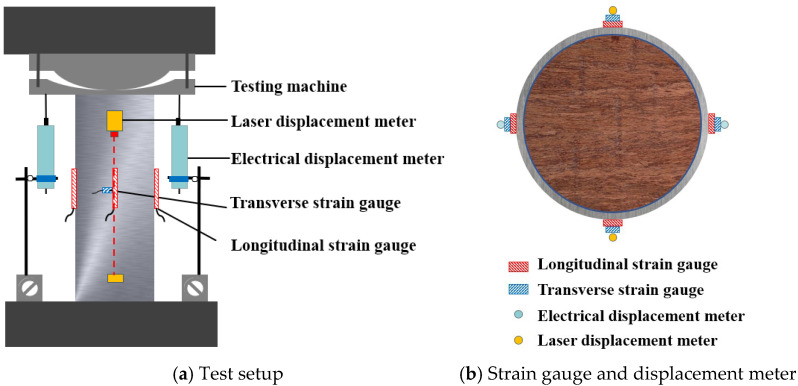
Test loading arrangement.

The loading device used in the test was a 3000 kN high-stiffness testing machine, and the TDS-530 data acquisition instrument, manufactured by TML Co., Ltd. in Tokyo, Japan, was used to collect the test strain, displacement, and other data at the same frequency, which is shown in Figure 2. Two electrical differential meters were arranged symmetrically and vertically on both sides of the specimen to measure the deformation of the specimen in the full height range, and two laser displacement meters were arranged symmetrically on the other two sides, both of which were located at a distance of 30 mm from the end. At the same time, four longitudinal strain gauges and four transverse strain gauges were symmetrically attached around the middle of each specimen. The test was conducted using displacement control at a rate of 0.5 mm/min. The test was terminated when the load decreased to 85% of the ultimate load or when the deformation reached 50 mm. For the core-loaded specimens, high-stiffness pads were placed on both ends of the specimens before loading, with a thickness of 4 mm and the same cross-section size as that of the core bamboo scrimber.

## 3. Results

### 3.1. Experimental Phenomena and Failure Modes

**Figure 3:**
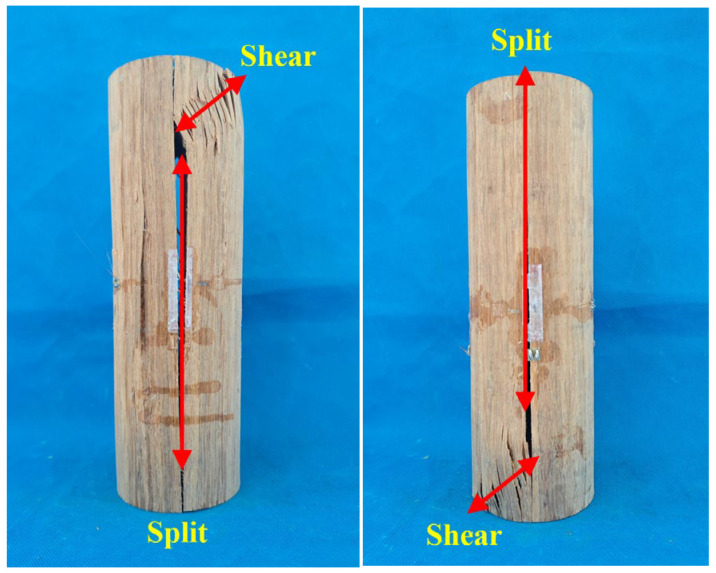
Failure modes of the bamboo scrimber columns.

Figure 3 illustrates the failure modes observed in bamboo scrimber columns, which include shear damage and splitting damage. When the bamboo scrimber column was subjected to compression, an oblique shear damage surface initially formed at the end of the specimen due to the combined effects of compressive and shear stresses. Additionally, the transverse fibers of the bamboo exhibited delamination as a result of the applied shear force. Since the bamboo scrimber column was constructed through the secondary cold-pressing and gluing of two semicircular cross-sections, cracks developed at the secondary gluing surface when the shear line extended to the center of the specimen. Ultimately, this led to the specimen splitting and losing its load-bearing capacity.

**Figure 4:**
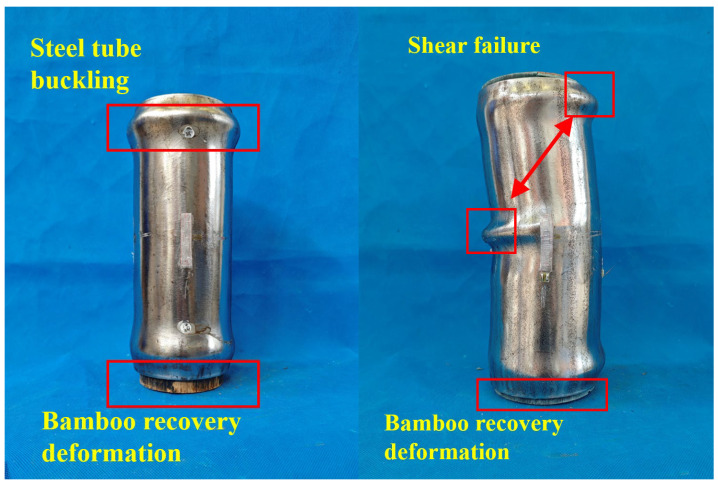
Failure modes of the composite columns under full-section loading.

Figure 4 illustrates the failure modes observed in full-section-loaded bamboo scrimber-filled steel tubular columns, which primarily exhibited buckling and shear damage. Some specimens near the end of the steel tube experienced localized buckling, ultimately resulting in a horizontal, ring-like bulge. Upon unloading, the plastic deformation of the bamboo was less pronounced than that of the steel tube. The bamboo component could even recover after it was stretched out from the steel tube. Additionally, the other specimens exhibited shear damage, with the local buckling of the steel tube occurring not in a horizontal plane but rather distributed along an oblique shear slip surface.

**Figure 5:**
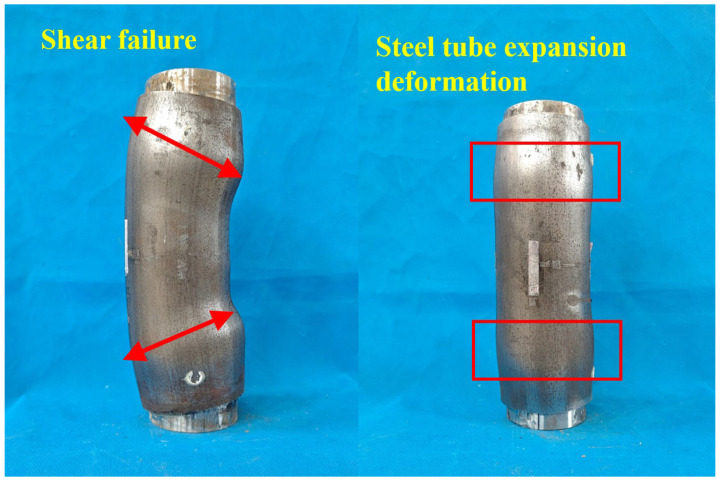
Failure modes of the composite columns under core loading.

Figure 5 illustrates the failure modes of the core-loaded composite column. The steel tube constrained the bamboo in the circumferential direction, while the pads exerted pressure on the bamboo. This interaction resulted in increased lateral deformation of the bamboo, which subsequently caused outward expansion and deformation of the steel tube. Since the steel tube was not subjected to vertical loads in the core-loading specimen, its buckling deformation was significantly less than that observed in the full-section-loading specimen. In the specimen with a steel tube thickness of 4 mm, the restraining effect of the steel tube on the bamboo scrimber was relatively weak, leading to noticeable internal shear damage under pressure. Additionally, distinct oblique shear surfaces could be observed on the surface of the steel tube.

### 3.2. Stress–Strain Relationship Curves

**Figure 6:**
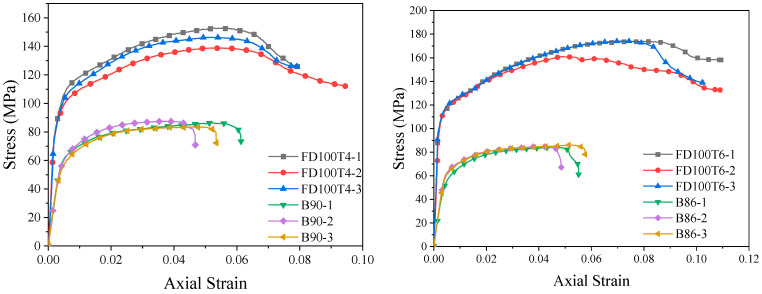
Stress–strain curves of BSFST columns under full-section-loading compression.

The stress–strain relationship curves of the full-section-loaded BSFST columns are illustrated in Figure 6. These curves can be primarily categorized into three stages: the elastic stage, the elastic–plastic stage, and the descending stage. During the elastic stage, both the steel tube and the bamboo scrimber share the load, resulting in a linear increase in the stress–strain curve. The initial stiffness is provided collaboratively by the bamboo scrimber and the steel tube, with the slope of the curve being steeper than that of the elastic section of the bamboo scrimber column. As the plastic properties of the steel tube and the bamboo scrimber column become more pronounced, the slope of the elastic–plastic curve gradually decreases, while the corresponding strain increases at a rate that surpasses that of the stress. Concurrently, the hoop force between the two materials subjects the steel tube to a state of longitudinal compression, hoop tension, and radial pressure. Consequently, the ring tensile stress in the steel tube gradually increases. According to the von Mises yield criterion, the longitudinal compressive stress in the steel tube decreases. In contrast, the bamboo scrimber experiences a state of three-way compression due to longitudinal pressure, hoop pressure, and radial pressure, leading to a significant increase in its compressive strength. The constraint provided by the steel tube results in a more uniform stress distribution within the bamboo scrimber, thereby reducing the likelihood of localized damage at the center of the bamboo. Once the composite structure reaches its peak stress, the stress of the specimen begins to decrease gradually, while the strain continues to increase until final failure occurs. The end point of the curve is defined as the limit point of the specimen, where the stress and strain are defined as the ultimate stress *f_u_* and ultimate strain *ε_u_* of the core-loaded specimen.

**Figure 7:**
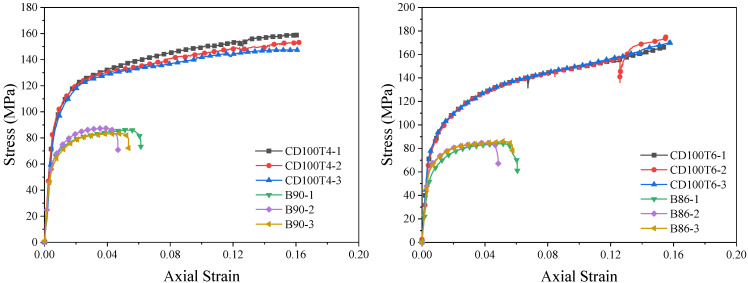
Stress–strain curves of BSFST columns under core-loading compression.

The stress–strain relationship curves of the BSFST columns under core loading can be divided into elastic and elastic–plastic stages, as illustrated in Figure 7. In the linear elastic stage, the outer steel tube exerts a minimal restraining effect on the bamboo scrimber. Consequently, the stress in the bamboo scrimber increases approximately linearly, with the initial stiffness being provided by the bamboo itself. The slope of the curve closely resembles that of the elastic section of the bamboo scrimber column. In the elastic–plastic stage, the slope of the stress–strain curve decreases, indicating that the constraint provided by the steel tube dominates the mechanical behavior of the specimen. The lateral pressure exerted by the steel tube restricts the transverse expansion and deformation of the bamboo scrimber, thereby inhibiting its splitting damage and effectively enhancing its compressive strength. In the late elastic–plastic stage, the curve exhibits a more stable secondary stiffness. Due to the limitations of the testing instrument, the subsequent stress–strain curve did not capture the descending section. Based on the trend of the curve, it can be inferred that the core-compressed specimen possesses greater compressive and deformation capacities.

### 3.3. Parameter Analysis

#### 3.3.1. Comparative Analysis of the Bamboo Scrimber Columns and BSFST Columns

**Figure 8:**
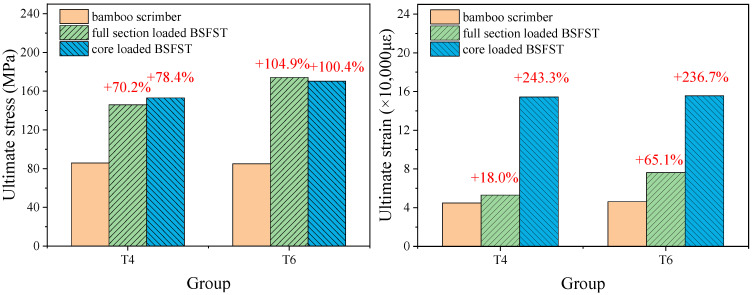
Comparison of the mechanical properties of the bamboo scrimber and BSFST columns.

As illustrated in Figure 8, the BSFST columns exhibited a significant increase in both ultimate stress and ultimate strain compared to the bamboo columns. For the full-section-loading specimens, the ultimate stresses increased by 70.2% and the ultimate strains increased by 18.0% for a steel tube thickness of 4 mm. In contrast, for a steel tube thickness of 6 mm, the ultimate stresses increased by 104.9% and the ultimate strains increased by 65.1%. For the core-loaded specimens, the ultimate stresses increased by 78.4% and the ultimate strains increased by 243.3% for the composite column specimens with a steel tube thickness of 4 mm. Similarly, for the composite column specimens with a steel tube thickness of 6 mm, the ultimate stresses increased by 100.4% and the ultimate strains increased by 236.7%. The steel tube restraint significantly enhances the load-carrying capacity and deformation capacity of the reconstituted bamboo. Both the full-section-loaded specimens and the core-loaded specimens demonstrate similar effects in enhancing load-carrying capacity; however, the core-loaded specimens exhibit a more pronounced effect in improving deformation capacity.

Analysis of variance (ANOVA) is a commonly used analytical method that can effectively eliminate the influence of intra-group data errors and determine whether there is a significant difference between two groups of data. In the analysis process, the *p*-value is calculated through the calculation method of the F-test, and the judgment is made by comparing it with 0.05. When *p* < 0.05, it indicates that there is a significant difference between the two groups of data, suggesting that the variable parameter indeed has a significant effect on the experimental results.

Through F-test calculations, the *p*-values for the ultimate stress and ultimate strain of the T4 series specimens in Figure 8 are 0.00000851 and 0.00000190, respectively; for the T6 series specimens, the *p*-values for ultimate stress and ultimate strain are 0.00000118 and 0.00000264, respectively. This indicates that there are significant differences in ultimate stress and ultimate strain between BSFST and bamboo columns. On this basis, further analysis using Tukey’s test shows that the *p*-values for the ultimate stress of BSFST columns under full-section loading and core loading are 0.246 and 0.921, both greater than 0.05; while the *p*-values for ultimate strain are 0.00000341 and 0.000792, both less than 0.05. This suggests that different loading modes have no significant effect on the ultimate stress of BSFST columns but have a significant effect on the ultimate strain.

#### 3.3.2. Influence of Steel Ratio

**Figure 9:**
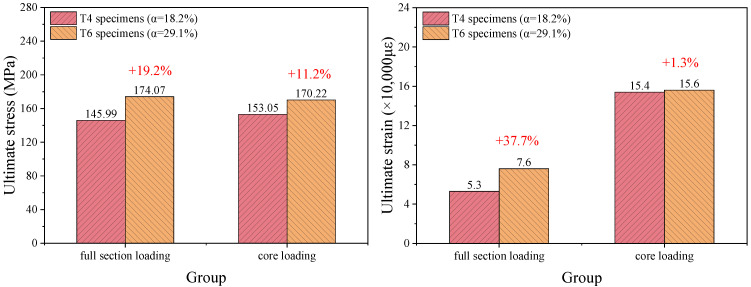
Comparison of the mechanical properties of the BSFST columns with different steel ratios.

As shown in Figure 9, the ultimate stress and strain of the BSFST columns increased with an increasing steel ratio. When the steel ratio increased from 18.2% to 29.1%, the ultimate stress and strain of the full-section-loaded specimens increased by 19.2% and 37.7%, respectively. For the core-loaded specimens, the increases were 11.2% and 1.3%, respectively. The steel ratio had a limited effect on the load-bearing capacity of the specimens. This is because the bamboo carried most of the longitudinal load in the full-section-loaded specimens, while in the core-loaded specimens, the steel tubes did not bear longitudinal loads but only provided lateral confinement. However, steel ratio significantly affected the deformation capacity of full-section-loaded specimens. This suggests that the steel confinement effectively delayed transverse fiber delamination and mitigated compression-induced splitting in the bamboo, thereby enhancing its mechanical performance.

Through F-test calculations, on the one hand, the *p*-values for the ultimate stress and ultimate strain of full-section-loaded specimens in Figure 9 are 0.0165 and 0.00356, respectively, both less than 0.05. This indicates that increasing the steel ratio can significantly improve the ultimate stress and strain for full-section-loaded specimens. On the other hand, the *p*-value for the ultimate stress of core-loaded specimens is 0.0139, which is less than 0.05, suggesting that the steel ratio has a significant impact on the bearing capacity of core-loaded specimens. However, the ultimate strain of core-loaded specimens corresponds to the maximum displacement at the termination of the test. Therefore, this ultimate strain value is a nominal value based on the test conditions rather than a true ultimate state, and it is meaningless to perform an analysis of variance on it.

#### 3.3.3. Influence of Loading Modes

**Figure 10:**
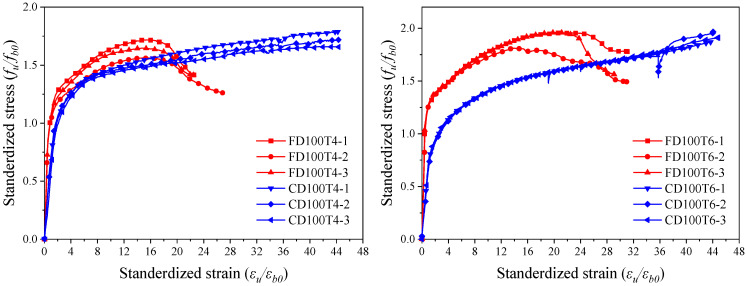
Comparisons of stress–strain curves of BSFST columns under different loading modes.

Figure 10 presents the normalized stress–strain curves of BSFST columns under different loading modes. The horizontal axis represents the standardized strain (*ε_u_*/*ε_b0_*), defined as the ratio of the specimen’s longitudinal strain to that of the standard bamboo scrimber specimen. The vertical axis represents the standardized stress (*f_u_*/*f_b0_*), defined as the ratio of the axial stress to the compressive strength of the standard bamboo scrimber specimen. Due to limitations in test conditions, the maximum measurable strain of the core-loaded specimens was 0.16. As a result, the descending branch of the curve was not captured.

The ultimate stresses of the full-section- and core-loaded specimens were similar, while the core-loaded specimens exhibited greater ultimate strain. As the core-loaded specimens were compressed solely through the bamboo scrimber, while both bamboo and steel contributed to compression in the full-section-loaded specimens, the initial stiffness of the latter was higher. Consequently, the elastic slope of their stress–strain curve was also steeper. The core-loaded specimens exhibited better load-bearing and deformation capacities. This is attributed to the fact that the steel tube in the core-loaded specimens was not subjected to axial compression and acted solely as a lateral confining element. As a result, the steel experienced a simpler stress state, avoiding early local buckling and thereby providing more effective confinement.

## 4. Discussion

### 4.1. Typical Stress–Strain Curves of BSFST Columns

**Figure 11:**
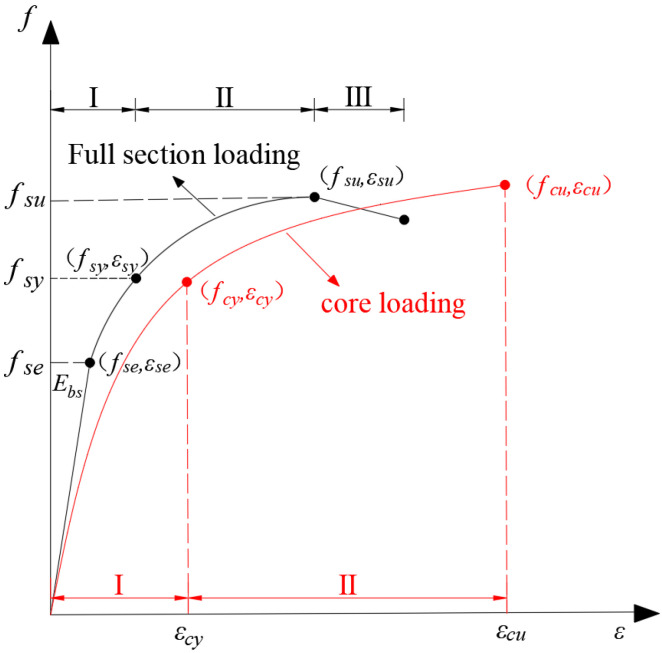
Typical stress–strain curves of BSFST columns under different loading modes.

From Figure 6 and Figure 7, we can observe that the stress–strain curves of the BSFST specimens exhibit certain characteristics and variation patterns. These variation patterns have been refined into the typical stress–strain curves of BSFST columns under different axial compression modes with reference to the constitutive relation curve of FRP-confined concrete [26], as shown in Figure 11. The stress–strain curves of the full-section-loaded specimens can be divided into three stages: elastic, elastic–plastic, and descending. The end of the elastic stage is defined as the elastic limit point, corresponding to the elastic limit stress and strain. The peak of the curve is defined as the ultimate point. The region between the elastic limit and ultimate points corresponds to the elastic–plastic stage, where the yield point is determined using the farthest point method [23]. The stress–strain curve of the core-loaded specimens consists of two stages: elastic and elastic–plastic. Since the descending branch was not recorded, the end of the curve is taken as the ultimate point. The yield point was also identified using the farthest point method.

However, the test results of the core compression specimens had certain limitations. Due to the constraints of the loading conditions, the maximum displacement under axial loading was within 50 cm. Therefore, when the maximum axial displacement was reached, the core compression specimens still had stable bearing capacity, and the curves still showed an upward trend, with the ultimate load failing to be measured. As a result, the curves of the core compression specimens were not complete. In subsequent studies, consideration will be given to further improving the test conditions to measure the complete stress–strain curves of the core compression specimens.

### 4.2. Calculation Models of the Full-Section-Loaded BSFST Columns

The bamboo-scrimber-filled steel tubular (BSFST) columns proposed in this paper share the same structural form as concrete-filled steel tubular (CFST) columns and FRP-confined concrete columns, and there are similarities in the calculation of ultimate strength that can be drawn upon. In the theory of concrete-filled steel tubular (CFST) and FRP-confined concrete columns [27], the calculation formula for ultimate strength is generally expressed as the compressive strength of plain concrete multiplied by an enhancement factor, which is related to the lateral confinement strength of concrete provided by steel tubes and FRP. The lateral confinement effect of steel tubes on concrete can be characterized by the hoop coefficient. The hoop coefficient *ξ_s_* is a key parameter influencing the ultimate bearing capacity, as it reflects the degree of lateral confinement provided by the steel tube. In this study, the specimens are bamboo scrimber columns confined by steel tubes. The hoop coefficient *ξ_s_* is incorporated into the theoretical model to evaluate the ultimate bearing capacity. However, in practical applications, the confinement effect of the steel tube is influenced by various factors, including the bond quality between the steel and bamboo, interface gaps, and dimensional variations of the steel tube.

(1) $$\xi_{s}=\frac{A_{s}f_{sy}}{A_{b}f_{b0}}=\alpha \frac{f_{sy}}{f_{b0}}$$

In the equation, *f_sy_* denotes the yield strength of the steel tube; *f_b__0_* denotes the standard axial compressive strength of the bamboo scrimber; α denotes the steel ratio.

Wu et al. [28] first proposed the concept of steel-tube-confined bamboo scrimber structures and conducted extensive experiments. The test results were analyzed to evaluate the ultimate bearing capacity of full-section-loaded bamboo columns. Based on the experimental analysis, a formula was developed to calculate the ultimate stress and strain of the full-section-loaded specimens, which are given as Equations (2) and (3). Building on this, coefficient corrections are proposed in this paper. The revised formula is applied to both the experimental data from this study and Wu et al.’s results [28].

(2) $$\frac{f_{u}}{f_{bu}}=1+1.047\xi_{s}$$

(3) $$\frac{\varepsilon_{u}}{\varepsilon_{bu}}=1+0.630\xi_{s}$$

### 4.3. Discussion

**Figure 12:**
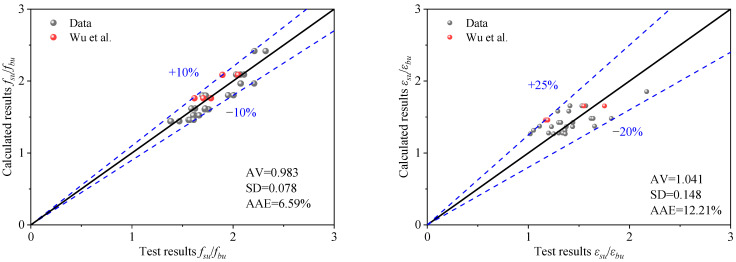
Evaluation of the proposed model for BSFST columns under full-section loading [28].

The comparison results indicate that the model predictions are in good agreement with the experimental values, as shown in Figure 12. The function of the black solid line is y = x, representing the ideal situation where the theoretical value is equal to the experimental value. The error range of the predicted data calculated by the proposed model is shown in the Figure 12 with the blue dashed line. For the ultimate stress model, the average value (AV), standard deviation (SD), and average absolute error (AAE) are 0.983, 0.078, and 6.59%, respectively. For the ultimate strain model, these values are 1.041, 0.148, and 12.21%, respectively. The errors between the experimental and predicted values are small, indicating that the proposed models offer high prediction accuracy. 

In fact, the specimens in this paper are short columns with a height-to-diameter ratio of 3:1, and their failure mode is mainly material failure. The prediction model of the full-section-loaded BSFST columns has certain limitations, as it is only applicable to short BSFST columns under pure axial compression. However, in practical engineering, the height-to-diameter ratios and stress conditions of columns are complex and variable. Long columns may undergo flexural–compressive failure under axial compression, and columns under eccentric compression are subject to both bending moment and axial force. Under these circumstances, the ultimate bearing capacity of BSFST columns will be reduced to a certain extent. Therefore, to better provide theoretical guidance for the engineering application of BSFST columns, studies on the axial compression and eccentric compression bearing capacities of long columns are essential in future research plans.

The full-section-loading mode mainly corresponds to most compression states of BSFST columns in engineering applications, while the research purpose of the core-loading mode is to explore the mechanical properties of reconstituted bamboo columns after being confined and strengthened by steel tubes during their service life. However, due to limitations in the test conditions, the curves of core-loaded specimens did not have a descending segment, and the maximum load was not measured. Therefore, this paper cannot provide an accurate prediction model for the ultimate bearing capacity of core-loaded BSFST columns, and only a qualitative analysis of their mechanical properties can be conducted. It is evident that the bearing capacity and deformation capacity of core-loaded specimens are superior to those of full-section-loaded specimens.

On the other hand, in Wu et al.’s test [29], the core-loading test was terminated when the displacement reached 30 mm, whereas in the present study, the test was terminated at a displacement of 50 mm. In both cases, the descending portion of the stress–strain curve was not captured, and the end of the curve was taken as the limit point. Since the limit point was defined differently in the two tests, the formula proposed by Wu et al. [29] for calculating the ultimate stress of the core-loaded specimens significantly underestimates the values observed in this study, indicating that it is not applicable to the data presented in this study. Similarly, the formula calibrated based on the data from this study is unlikely to be applicable to Wu et al.’s [29] results. Therefore, no formula for the ultimate stress and strain of core-loaded specimens is proposed in this study.

## 5. Conclusions

In this study, 18 BSFST columns and bamboo scrimber columns were axially loaded under full-section- and core-loading conditions. Critical variables, including the steel ratio and the loading mode, were systematically analyzed. The conclusions are drawn as follows:

(1) The primary failure modes of bamboo-scrimber-filled steel tubular columns under axial compression are shear failure and buckling. For the full-section-loaded specimens, evident local buckling, ring-like bulging, and inclined shear cracks were observed on the steel tube surface. For the core-loaded specimens, the steel tube showed outward bulging with little buckling. Specimens with a tube thickness of 4 mm exhibited pronounced shear failure on the surface.
(2) The stress–strain curve of a full-section-loaded BSFST column can be divided into three stages, namely elastic, elastic–plastic, and descending stage. For the core-loaded specimens, the stress–strain curve includes two stages, namely elastic and elastic–plastic. With an increasing steel ratio, the elastic stage becomes longer and the slope of the elastic–plastic stage increases, indicating a significant enhancement in both load-bearing and deformation capacity. With the steel ratio increasing, the ultimate stress of the specimen rises up to 19.2%, while the ultimate strain increases by as much as 37.7%.
(3) The ultimate stresses of the core-loaded and full-section-loaded BSFST specimens are comparable, while the core-loaded specimens exhibit higher ultimate strains. Under core loading, only the bamboo bears the axial load exclusively, while the steel tube functions solely as lateral confinement. In contrast, in full-section-loaded specimens, the steel tube is subjected to direct axial compression and tends to yield prematurely, leading to reduced confinement effectiveness and limited overall deformability.
(4) The existing prediction model for the ultimate stress and strain of BSFST columns was modified by incorporating the hoop confinement coefficient of the steel tube. By validating with both current and previously published experimental data, the proposed modified model characterizes a good agreement between the predicted and experimental results, indicating an excellent prediction for future engineering applications.

## Figures and Tables

**Table 1:** Parameters and test results of the specimens.

Specimens	*D* (mm)	*T* (mm)	*α*	*P_u_* (kN)	*f_y_* (MPa)	*ε_y_*	*f_u_* (MPa)	*ε_u_*
B86-1	86	/	/	530.49	81.02	0.0269	83.99	0.0468
B86-2	86	/	/	536.40	81.71	0.0238	84.74	0.0405
B86-3	86	/	/	543.87	83.07	0.0291	86.11	0.0514
B90-1	90	/	/	547.07	83.33	0.0352	86.42	0.0518
B90-2	90	/	/	553.52	84.41	0.0224	87.44	0.0369
B90-3	90	/	/	528.77	80.61	0.0243	83.53	0.0461
FD100T4-1	100	4	18.2%	1199.85	110.90	0.0061	152.85	0.0525
FD100T4-2	100	4	18.2%	1089.66	102.29	0.0060	138.81	0.0530
FD100T4-3	100	4	18.2%	1148.57	107.29	0.0063	146.31	0.0536
FD100T6-1	100	6	29.1%	1365.47	116.04	0.0046	173.95	0.0810
FD100T6-2	100	6	29.1%	1263.40	116.92	0.0046	160.94	0.0487
FD100T6-3	100	6	29.1%	1367.49	118.46	0.0047	174.20	0.0716
CD100T4-1	100	4	18.2%	1055.57	122.08	0.0209	158.87	0.1557
CD100T4-2	100	4	18.2%	1014.64	118.07	0.0179	152.71	0.1559
CD100T4-3	100	4	18.2%	980.56	119.76	0.0214	147.58	0.1512
CD100T6-1	100	6	29.1%	1009.85	116.01	0.0249	166.12	0.1537
CD100T6-2	100	6	29.1%	1062.13	118.03	0.0273	174.72	0.1551
CD100T6-3	100	6	29.1%	1032.40	119.05	0.0289	169.83	0.1579

Note: Specimens were numbered according to the different parameters of the specimens; *α*—steel ratio, which equals *A_s_*/*A_b_* × 100%; *P_u_*—ultimate bearing capacity; *f_u_*—ultimate stress, for full-section-loading specimens, *f_u_* = *P_u_*/(*A_s_* + *A_b_*), for core-loading specimens, *f_u_* = *P_u_*/*A_b_*; *ε_u_*—ultimate strain, corresponding to the ultimate stress; *f_y_* and *ε_y_* are the yield stress and strain, which were determined by the farthest point method [23].

**Table 2:** Mechanical properties of steel tube.

Steel Tube	Yield Stress (MPa)	Ultimate Stress (MPa)	Elastic Modulus (GPa)
T4	356.15	489.52	205.96
T6	316.35	469.76	205.47

**Table 3:** Mechanical properties of bamboo scrimber.

Tension			Compression		
Ultimate Stress (MPa)	Ultimate Strain	Elastic Modulus (GPa)	Ultimate Stress (MPa)	Ultimate Strain	Elastic Modulus (GPa)
154.32	0.0084	18.50	88.88	0.0352	16.91

## Data Availability

The original contributions presented in this study are included in the article. Further inquiries can be directed to the corresponding authors.
